# Intensified Adjuvant IFADIC Chemotherapy for Adult Soft Tissue Sarcoma: A Prospective Randomized Feasibility Trial

**DOI:** 10.1155/2000/126837

**Published:** 2000-12-22

**Authors:** Thomas Brodowicz, Eva Schwameis, Joachim Widder, Gabriele Amann, Christoph Wiltschke, Martin Dominkus, Reinhard Windhager, Peter Ritschl, Richard Pötter, Rainer Kotz, Christoph C. Zielinski

**Affiliations:** ^1^ Clinical Division of Oncology, Vienna, Austria; ^2^ Department of Orthopedic Surgery, Vienna, Austria; ^3^ Department of Radiotherapy and Radiobiology, Vienna, Austria; ^4^ Department of Clinical Pathology, University Hospital, Vienna, Austria; ^5^ Department of Orthopedic Surgery, Orthopedic Hospital Gersthof, Vienna, Austria; ^6^ Medical Experimental Oncology and Ludwig Boltzmann Institute for Clinical Experimental Oncology, Department of Medicine I, University Hospital, 18-20 Waehringer Guertel, Vienna A-1090, Austria

## Abstract

*Purpose*. The present prospective randomized adjuvant trial was carried out to compare the toxicity, feasibility and efficacy of augmented chemotherapy added to hyperfractionated accelerated radiotherapy after wide or marginal resection of grade 2 and grade 3 soft tissue sarcoma (STS).

*Patients and methods*. Fifty-nine patients underwent primary surgery by wide or marginal excision and were subsequently randomized to receive radiotherapy alone or under the addition of six courses of ifosfamide (1500 mg/m2 , days 1–4), dacarbazine (DTIC) (200 mg/m2 , days 1–4) and doxorubicin (25 mg/m2 , days 1–2) administered in 14-day-intervals supported by granulocyte-colony stimulating factor (30 × 106 IU/day, s.c.) on days 5–13. According to the randomization protocol, 28 patients received radiotherapy only, whereas 31 patients were treated with additional chemotherapy.

*Results*. The relative ifosfamide–doxorubicin–DTIC (IFADIC) dose intensity achieved was 93%. After a mean observation period of 41±19.7 months (range, 8.1–84 months), 16 patients (57%) in the control group versus 24 patients (77%) in the chemotherapy group were free of disease (p>0.05).Within the control group, tumor relapses occurred in 12 patients (43%;six patients with distant metastases, two with local relapse, four with both) versus seven patients (23%; five patients with distant metastases, one with local recurrence, one with both) from the chemotherapy group. Relapse-free survival (RFS) (p=0.1), time to local failure (TLF) (p=0.09), time to distant failure (TDF) (p=0.17) as well as overall survival (OS) (p=0.4) did not differ significantly between the two treatment groups. Treatment-related toxicity was generally mild in both treatment arms.

*Conclusions*. We conclude that the safety profile of intensified IFADIC added to radiotherapy was manageable and tolerable in the current setting. Inclusion of intensified IFADIC was not translated into a significant benefit concerning OS, RFS, TLF andTDF as compared with radiotherapy only, although a potential benefit of chemotherapy for grade 3 STS patients needs to be validated in prospective randomized trials including larger patient numbers.


*Sarcoma* (2000) **4**, 151–160

ORIGINAL ARTICLE

### Intensified adjuvant IFADIC chemotherapy for adult soft tissue sarcoma: a prospective randomized feasibility trial

THOMAS BRODOWICZ,[1] EVA SCHWAMEIS,[2] JOACHIM WIDDER,[3] GABRIELE AMANN,[4] CHRISTOPH WILTSCHKE,[1] MARTIN DOMINKUS,[2] REINHARD WINDHAGER,[2] PETER RITSCHL,[5] RICHARD PÖTTER,[3] RAINER KOTZ[2] & CHRISTOPH C. ZIELINSKI,[1,6] FOR THE AUSTRIAN COOPERATIVE SOFT TISSUE SARCOMA STUDY GROUP*

*[1]Clinical Division of Oncology, [6]Chair for Medical Experimental Oncology and Ludwig Boltzmann Institute for Clinical Experimental Oncology, Department of Medicine I, [2]Department of Orthopedic Surgery, [3]Department of Radiotherapy and Radiobiology, [4]Department of Clinical Pathology, University Hospital, and [5]Department of Orthopedic Surgery, Orthopedic Hospital Gersthof; Vienna, Austria*

**Abstract**

*Purpose.* The present prospective randomized adjuvant trial was carried out to compare the toxicity, feasibility and efficacy of augmented chemotherapy added to hyperfractionated accelerated radiotherapy after wide or marginal resection of grade 2 and grade 3 soft tissue sarcoma (STS).

*Patients and methods.* Fifty-nine patients underwent primary surgery by wide or marginal excision and were subsequently randomized to receive radiotherapy alone or under the addition of six courses of ifosfamide (1500 mg/m$^2$, days 1–4), dacarbazine (DTIC) (200 mg/m$^2$, days 1–4) and doxorubicin (25 mg/m$^2$, days 1–2) administered in 14-day-intervals supported by granulocyte-colony stimulating factor ($30 \times 10^6$ IU/day, s.c.) on days 5–13. According to the randomization protocol, 28 patients received radiotherapy only, whereas 31 patients were treated with additional chemotherapy.

*Results.* The relative ifosfamide–doxorubicin–DTIC (IFADIC) dose intensity achieved was 93%. After a mean observation period of 41±19.7 months (range, 8.1–84 months), 16 patients (57%) in the control group versus 24 patients (77%) in the chemotherapy group were free of disease ($p$>0.05). Within the control group, tumor relapses occurred in 12 patients (43%; six patients with distant metastases, two with local relapse, four with both) versus seven patients (23%; five patients with distant metastases, one with local recurrence, one with both) from the chemotherapy group. Relapse-free survival (RFS) ($p$=0.1), time to local failure (TLF) ($p$=0.09), time to distant failure (TDF) ($p$=0.17) as well as overall survival (OS) ($p$=0.4) did not differ significantly between the two treatment groups. Treatment-related toxicity was generally mild in both treatment arms.

*Conclusion.* We conclude that the safety profile of intensified IFADIC added to radiotherapy was manageable and tolerable in the current setting. Inclusion of intensified IFADIC was not translated into a significant benefit concerning OS, RFS, TLF and TDF as compared with radiotherapy only, although a potential benefit of chemotherapy for grade 3 STS patients needs to be validated in prospective randomized trials including larger patient numbers.

**Key words**: *adjuvant, chemotherapy, radiotherapy, sarcoma, soft tissue*

### Introduction

Patients with high-grade soft tissue sarcomas (STS) treated by local tumor removal remain free of relapse in approximately 40–60%, whereas overall survival rates range between 50 and 70%.[1–3] As surgical resection represents the mainstay of treatment in adult extremity STS, the outcome depends on high-quality preoperative tumor imaging followed by an optimal surgical approach that should keep an equilibrium between complete removal of malignancy and a minimum of functional impairment. The use of adjuvant radiotherapy (RT) after wide or compartmental surgery of STS varies between centers, and in some geographic areas is restricted to patients with histopathologic intermediate- or high-grade STS.[4,5] Other centers apply adjuvant RT regardless of surgical margins and malignancy grade.[6,7] However, an area including the complete extension of the initial tumor with its risk zone and the entire scar need to be treated. In an attempt to reduce the rate of local relapses, hyperfractionated RT has emerged as an attractive possibility in the treatment of sarcomas.[8] However, despite refinements in local therapy, distant

Correspondence to: Christoph C. Zielinski, M.D., Professor of Medicine and Medical Experimental Oncology, Department of Medicine I, University Hospital, 18-20 Waehringer Guertel, A-1090 Vienna, Austra. Tel: 43/1/40400-4445; Fax: 43/1/40400-4452; E-mail: christoph.zielinski@akh-wien.ac.at

1357-714X print/1369-1643 online/00/040151-10 © 2000 Taylor & Francis Ltd

*DOI: 10.1080/13577140020025869*

metastases occur in about 50% of adult patients with high-grade STS.[9] Consequently, prospective randomized studies on the efficacy of adjuvant chemotherapy (CT) have been carried out, and resulted in the demonstration of a favorable effect upon both relapse-free (RFS) and overall survival (OS) in three studies,[10–13] whereas three further studies showed a significant benefit for RFS only.[14–16] Finally, a recently published meta-analysis showed an absolute improvement for patients with STS resulting from the administration of adjuvant CT concerning overall (absolute benefit 10%), local (absolute benefit 6%) and distant recurrence-free survival (absolute benefit 10%) at 10 years.[17]

Cytotoxic agents most frequently used in polychemotherapy for STS include doxorubicin, ifosfamide, cyclophosphamide and DTIC. The Southwestern Oncology Group has reported on a 50% response rate of recurrent/metastatic STS following the administration of a combination of doxorubicin, DTIC, cyclophosphamide and vincristine (CYVADIC),[18] whereas another chemotherapeutic combination used for this indication has been put together to include agents with the highest single-agent activity in adult STS,[19] including ifosfamide, doxorubicin and DTIC (IFADIC).[20,21]

Ever since the concept of dose intensity additionally defining efficacy of chemotherapy[22] has emerged, several trials testing this hypothesis have been published.[23] However, despite the lack of clear-cut data on the efficacy of adjuvant CT in STS, only few trials have attempted to improve results of this treatment modality by augmenting the dose of cytotoxic agents.[13,15,24,25] As an augmentation of dose can be achieved by either an increase in the amount of drug(s) administered or by an abbreviation of time intervals between treatment courses,[26] we used the latter approach and administered the IFADIC regimen in 14-day intervals with granulocyte-colony stimulating factor (G-CSF) support.

We now report the results of a prospective adjuvant feasibility trial performed in patients with STS after wide or marginal resection of malignancy and subsequent hyperfractionated accelerated RT randomizing between no further treatment and additional augmented CT with IFADIC. After a mean observation period of 41±19.7 months (range, 8.1–84 months), no significant differences in the duration of RFS, time to local failure (TLF) as well as time to distant failure (TDF) or OS ($p$>0.05) between the two treatment groups have emerged. The safety profile of intensified CT with IFADIC added to radiotherapy was manageable and tolerable.

### Patients and methods

### *Patients*

The study was initiated in January 1992 and conducted according to the declaration of Helsinki after having been approved by the local ethical committee. After a mean observation period of 41±19.7 months (range, 8.1–84 months), 59 STS patients (27 females, 32 males) with a mean age of 52 years (range, 20–77 years) were included. Characteristics of patients are presented in Table 1. Primary endpoints were treatment-related toxicities and dose intensity. RFS, TLF, TDF and OS constituted secondary endpoints.

**Table 1**. *Patients' characteristics*

| | RT and CT ($n$=31) | RT ($n$=28) |
|---|---|---|
| Characteristics | | |
| Mean age (years)±SD (range) | 49±14.5 (20–71) | 54±15.4 (21–77) |
| Female/male | 13/18 | 14/14 |
| Mean tumor size (cm)±SD (range) | 9.4±4.9 (2.5–20) | 8.8±4.2 (1–18) |
| Histology | | |
| Liposarcoma | 6 | 9 |
| MFH | 6 | 5 |
| Synovial sarcoma | 4 | 3 |
| Leiomyosarcoma | 6 | 0 |
| Malignant schwannoma | 1 | 0 |
| Fibrosarcoma | 2 | 2 |
| Rhabdomyosarcoma | 0 | 2 |
| Other types of STS | 6 | 7 |
| Tumor location | | |
| Upper extremity | 9 | 2 |
| Lower extremity | 16 | 20 |
| Trunk | 5 | 6 |
| Retroperitoneum | 1 | 0 |
| Tumor grading | | |
| G2 | 6 | 12 |
| G3 | 25 | 16 |

CT, Chemotherapy; MFH, malignant fibrous histiocytoma; RT, radiotherapy; STS, soft tissue sarcoma.

### *Inclusion criteria*

Inclusion criteria consisted of histopathologically verified grade 2 (tumor size >5 cm) or grade 3 (any tumor size) STS, performance status World Health Organization (WHO) 0–1 (=Karnofsky ≥60), an age of 18–80 years, serum total bilirubin and/or transaminase levels ≤1.25 times the upper limits of normal, serum creatinine ≤2 mg/100 ml, and an adequate hematologic function (as defined by white blood cells ≥3.0×$10^9$/l, platelets ≥100×$10^9$/l). Histologic entities included fibrosarcoma, malignant fibrous histiocytoma, liposarcoma, leiomyosarcoma, rhabdomyosarcoma, synovial sarcoma, malignant schwannoma, epitheloid sarcoma, clear cell sarcoma and mixed tumors of soft tissue origin.

### *Exclusion criteria*

Exclusion criteria consisted of previous chemo- or radiotherapeutic treatment of the current disease, intralesional resection of the primary tumor (see 'Treatment protocol' section), local relapse of previous STS, the presence of distant metastases at time of diagnosis, surgical resection being carried out >4 weeks before randomization, second malignancy with the exception of *in situ* cervical cancer or adequately excised basal cell or squamous cell carcinoma of the skin, left ventricular ejection fraction ≤50%, history of atrial or ventricular arrhythmias, histologic entities including neuroblastoma, primitive neuroectodermal tumor (PNET), Ewing sarcoma, extraskeletal osteosarcoma and embryonal rhabdomyosarcoma, active infection or any other serious underlying medical condition that would impair the ability of the patient to receive treatment according to the protocol, altered mental status that would prohibit the understanding and giving of informed consent, pregnancy and breast feeding.

### *Randomization and stratification*

Patients after wide or marginal surgical resection of histopathologically verified STS were randomized to receive either RT only or a combination of RT and CT. Stratification was carried out according to, first, location of the tumor (extremities versus trunk), second, age (≤45 years versus 46–80 years) and, third, the randomizing center.

### *Treatment protocol*

*Surgery and pathohistologic work-up. Surgery.* To achieve local tumor control, adequate surgery with tumor-free resection margins was required, representing the most important prerequisite. Thus, only patients after marginal or wide resection of STS were included in this study. Preoperative planning was performed after staging with computerized tomography and/or magnetic resonance imaging of the tumor region and, in case of involvement of vessels or nerves, angiography. Primary resection was performed with respect to adequate resection margins. To achieve limb salvage, endoprostheses were used to replace the resected bone if necessary. Moreover, reconstruction of vessels and nerves, and plastic surgery using free flaps or rotational flaps and skin grafts to cover the defect were performed. Resection margins were evaluated according to Enneking:[27] wide resection was defined as *en bloc* excision performed through healthy tissue beyond the reactive zone. Resection was defined as marginal if the resection was carried out extracapsularly but through the reaction zone surrounding the tumor. Patients with intralesional (intratumoral) resections were excluded.[28]

*Histopathologic analysis.* Central histopathology review was a prerequisite for inclusion of patients and was performed by Prof. M. Salzer-Kuntschik, Dr G. Amann and Dr S. Lang from the Department of Clinical Pathology at the University Hospital of Vienna. Acting as referral pathologists, histopathologic diagnosis was required to be made by at least two pathologists independently and with identical outcome. Both tumor classification and grading was performed according to Enzinger and Weiss, and Fletcher.[29,30] Grading was based on the modified scheme from Coindre using the following three parameters: (1) degree of tumor differentiation, with scores 1–3 including clearly recognizable entities such as alveolar soft part sarcoma in the group with score 2; (2) tumor necrosis, with scores 0–2; and (3) mitotic count with scores 1–3 corresponding to <10, <20 and ≥20 mitosis per 10 high-power fields.

Grade 1 tumors were thereby defined by a total score of ≤3. Grade 2 included tumors with a total score of 4 and 5. A total score of ≥6 defined grade 3 malignancy.

*Adjuvant augmented chemotherapy.* Ifosfamide (1500 mg/m$^2$, days 1–4), DTIC (200 mg/m$^2$, days 1–4) and doxorubicin (25 mg/m$^2$, days 1 and 2; IFADIC) were administered intravenously in a 14-day-cycle. G-CSF (Neupogen, Roche) (30×$10^6$ IU/day) was injected subcutaneously on days 5–13. The first course of chemotherapy started within 28 days following STS resection, and a total of six cycles of chemotherapy was given. In the case of randomization into the chemotherapy arm, radiotherapy was administered during cycles 3 and 4. Cycles 3 and 4 contained no doxorubicin due to concomitant radiotherapy.

*Supportive therapy.* Standard anti-emetic medication consisted of 5-HT$_3$-antagonists and metoclopramide. Hydration was carried out as indicated. Mesna uroprotection at 300 mg/m$^2$ i.v. was given 30 minutes before and 4 and 8 hours after ifosfamide. Substitution of oral mesna at 600 mg/m$^2$ was allowed for the 4- and 8-hour i.v. doses.

*Adjuvant hyperfractionated accelerated radiotherapy.* Radiotherapy consisted of 51 Gy within 3 weeks to be administered in single fractions of 1.7 Gy twice daily with an interfraction interval of at least 6 hours. The target volume encompassed the compartment of the primary tumor location and included the entire scar. Dose was calculated at the ICRU point. If the spinal cord, small intestine, or lung was in the field, conventional fractionation to a total dose of 50–60 Gy was to be administered.

*Evaluation of patients.* Before randomization was carried out, patients were staged according to the TNM classification for STS. In addition, the following procedures were performed. Obligatory: physical examination, laboratory tests for hematologic assessment and blood chemistry, chest X-ray, abdominal ultrasound, total body bone scintigraphy, computerized tomography or magnetic resonance imaging of the previous tumor bed and, finally, histopathologic examination including identification of type of malignancy and histopathologic grading.

Hematologic as well as non-hematologic toxicities were assessed according to WHO criteria on day 1 of each chemotherapy cycle. Local toxicity was assessed at the end of radiotherapy. Chronic local toxicity was assessed during follow-up evaluations. After completing treatment, the patients' status was assessed in intervals of 4 months and included physical examination, laboratory tests, chest X-ray, abdominal ultrasound and computerized tomography or magnetic resonance imaging of the previous tumor bed.

*Dose modifications.* Cytotoxic drug administration was delayed by 1 week in case of no full hematologic recovery (white blood cells $\geq 3.0 \times 10^9$/l, platelets $\geq 100 \times 10^9$/l) from the prior chemotherapy cycle.

*Dose intensity.*[31] Treatment duration was calculated as time between the first and final cytotoxic drug administration plus 14 days, representing the theoretical duration of one treatment cycle. The total administered dose represented the sum of all administered doses per square meter. Relative dose intensity was calculated by dividing the total administered dose in milligrams by total duration in days, and subsequently expressed as the percentage of initially planned theoretical dose intensity.

*Statistical analysis*

Relapse-free and overall survival were secondary end points for the study. Data are given as mean±standard deviation. Statistical calculations were carried out by log-rank test (overall survival, relapse-free survival, time to local failure, time to distant failure) or chi-square test (response rates, toxicity), both performed with the BMDP-PC program package using a level of significance of 0.05 (two-sided). Data were analyzed on an intent-to-treat basis. Fifty patients/treatment arm were aimed to be included in this study over an expected time period of 48 months. However, accrual was lower than expected and subsequently closed after the inclusion of a total of 59 patients. For relapse-free survival analysis, patients were considered to have failed treatment at relapse or death and were censored at the date of last contact if alive without relapse. For analyses of time to distant or local failure, patients were considered to have failed treatment at occurrence of distant metastases or at local recurrence, and were censored at the date of last contact or death without distant or local recurrence.

### Results

*Treatment-associated toxicity*

*Systemic toxicity of adjuvant augmented chemotherapy+ hyperfractionated accelerated radiotherapy.* The mean number of administered courses of chemotherapy was 5.7±1 (range, 2–6). Treatment-related toxicity in patients receiving chemotherapy was generally mild. A detailed description of toxicities is presented in Table 2. No systemic toxicity was observed in patients receiving radiotherapy only.

In detail, treatment-associated toxicity in patients from the chemotherapy group included alopecia of WHO grade 3 in all cases, leukopenia of WHO grades 1 and 2 in 19 patients (61%), grade 3 in four patients (13%) and grade 4 in four patients (13%), thrombocytopenia grades 1 and 2 in seven patients (23%), grade 3 in one patient (3%) and grade 4 in one

**Table 2.** *Treatment-associated systemic toxicity in 31 patients receiving adjuvant radiotherapy+adjuvant chemotherapy*

| | WHO grade | | | |
|---|---|---|---|---|
| | I | II | III | IV |
| Leukopenia | 11 (35%) | 8 (26%) | 4 (13%) | 4 (13%) |
| Thrombocytopenia | 5 (16%) | 2 (6%) | 1 (3%) | 1 (3%) |
| Alopecia | 0 | 0 | 31 (100%) | 0 |

WHO, World Health Organization.

patient (3%). Non-hematologic toxicity consisted of stomatitis WHO grade 3 in 1 patient (3%). Due to standard anti-emetic medication with 5-$HT_3$-antagonists combined with metoclopramide, no episodes of emesis grades III or IV occurred. Furthermore, none of the patients developed neutropenic sepsis. In two patients (6%), chemotherapy was discontinued after two cycles due to impairment of wound healing.

*Local toxicity of radiotherapy.* Generally, acute local toxicity from radiotherapy was mild. Most patients showed an erythema and dry desquamation; two (control group) versus three (chemotherapy group) patients had a moist desquamation. Local toxicities were individual in nature: no excessive fibrosis with considerable functional impairment was found. One infected endoprosthesis had to be removed in each group, respectively. One patient suffered from a fracture of his irradiated thigh, which occurred simultaneously with marked fibrosis and lymphedema 3 years after treatment (chemotherapy group). One last patient underwent limb amputation 7 months after treatment due to recurring fistulae and bone necrosis in the absence of tumor recurrence (chemotherapy group).

### Dose intensity

Patients randomized to the chemotherapy arm received a mean number of 5.7±1 (range, 2–6) chemotherapy cycles, all of which were given in the assigned dose. CT was discontinued in two patients after two cycles due to wound healing impairment. Thus, a total of 178 cycles were administered. Treatment was delayed in 21 cycles (11.8% of cycles, 19.4% of patients) due to hematologic toxicity. No cycle was delayed for >2 weeks and no dose reductions were necessary. Mean duration of treatment was 86.5±20.7 days (range, 28133 days). Mean administered doses of ifosfamide, DTIC and doxorubicin were 34452±5994 mg/m$^2$ (range, 12 000–36 000 mg/m$^2$), 4594±799 mg/m$^2$ (range, 1600–4800 mg/m$^2$) and 194±25 mg/m$^2$ (range, 100–200 mg/m$^2$), respectively. Relative dose intensity was 93%.

### Response to treatment

*RFS, TLF, TDF and OS.* After a mean follow-up of 41±19.7 months (range, 8.1–84 months), 16 patients (57%) from the control group versus 24 patients (77%) from the chemotherapy group had no evidence of tumor recurrence (Table 3). In contrast, recurrent disease developed in 12 patients (43%) treated with radiotherapy alone versus seven (23%) patients who received additional chemotherapy ($p$>0.05) (Table 3). The median dose of radiotherapy was 51 Gy (mean, 50 Gy) and median duration of radiotherapy was 21 days (mean, 27 days) with no difference between randomization groups ($p$>0.1).

*Sites of relapses.* The sites of STS relapses were as follows (Table 3). In the control group, six patients presented with lung metastases, two patients with local recurrence and four patients with both. In the chemotherapy group, five patients developed lung metastases, one patient local recurrence and one patient local relapse and lung metastases. Local failures occurred within the first 3 years following surgical removal of the primary tumor, whereas distant failures occurred up to 3.6 years after surgery. Overall, four patients died: one patient from each treatment group due to tumor progression, and two additional patients from the control group due to myocardial infarction that occurred 1 and 13 months after wide surgical resection of malignant fibrous histiocytoma of the left leg, respectively. As shown in a Kaplan–Meier analysis, RFS ($p$=0.1) (Fig. 1b), TLF ($p$=0.09) (Fig. 1c), TDF ($p$=0.17) (Fig. 1d) as well as OS ($p$=0.4) (Fig. 1a) did not differ significantly between the two treatment groups. However, patients randomized to receive chemotherapy tended to develop less local failures, as compared with patients receiving radiotherapy only ($p$=0.09). In a subgroup analysis, tumor size and histopathologic grading were analyzed and put into relation with response to treatment. Whereas tumor size was well balanced and without impact upon response to treatment (Table 4), histologic grading emerged to be of significant importance upon response to treatment concerning variables of disease outcome.

**Table 3.** *Responses to treatment*

| Reponse rates | RT and CT ($n$=31) | RT ($n$=28) |
|---|---|---|
| No evidence of tumor recurrence (%) | 24 (77%) | 16 (57%) |
| Overall relapses (%) | 7 (23%) | 12 (43%) |
| Local failures (%) | 2 (6%)[1] | 6 (21%)[2] |
| Distant failures (%) | 6 (19%)[1] | 10 (36%)[2] |

[1]Including one patient with local relapse and lung metastases.
[2]Including four patients with local relapse and lung metastases.
CT, Chemotherapy; RT, radiotherapy.

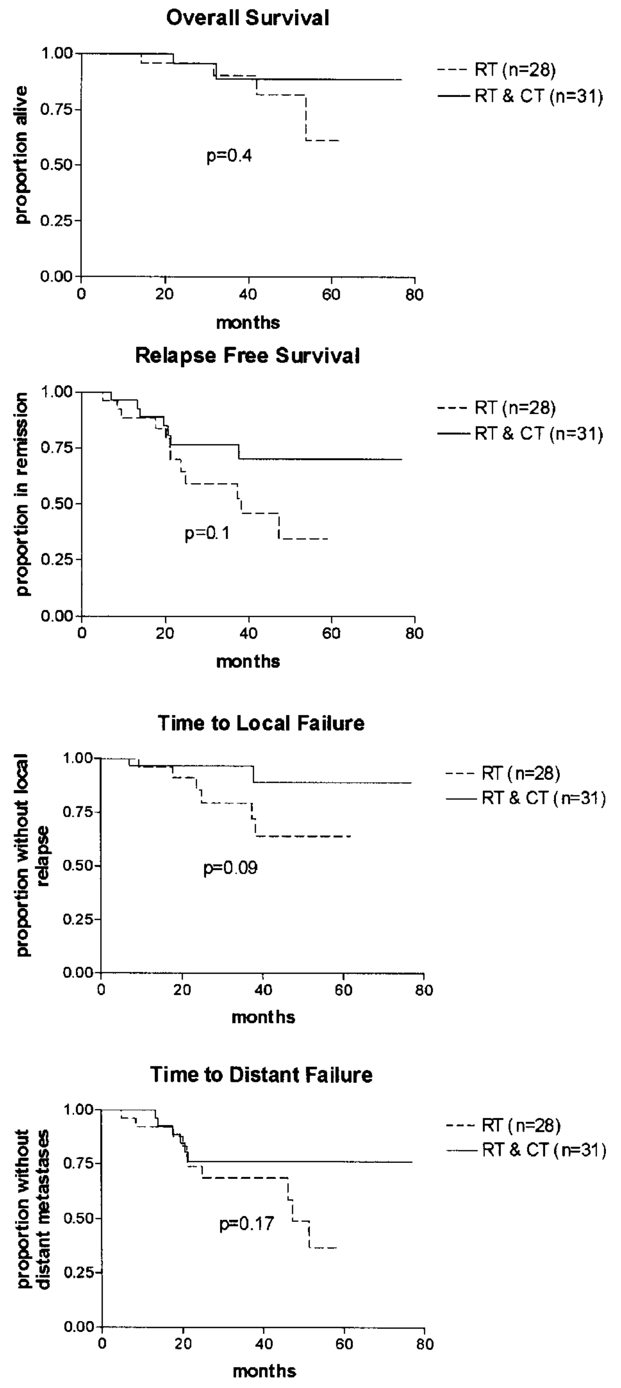

Figure 1.

*RFS, TLF, TDF and OS in patients with grade 3 STS.* Subgroup analysis of patients with grade 3 STS versus patients with grade 2 STS was carried out: 16 patients with grade 3 STS received radiotherapy only versus 25 patients who underwent additional chemotherapy. Seven patients (44%) from the control group versus 19 patients (76%) from the chemotherapy group had no evidence of tumor recurrence (Table 4). Tumor relapse occurred in nine patients (56%) treated with radiotherapy alone versus six (24%) patients who received additional chemotherapy ($p$<0.05, two-sided) (Table 4).

Sites of tumor recurrences were as follows (Table 4). In the control group, eight patients (50%) presented with lung metastases, including four who had additional local relapse. One further patient had local relapse alone. In contrast, in the chemotherapy group, five patients (20%; $p$<0.05, two-sided) developed lung metastases, including one patient with additional local relapse. One further patient presented with local recurrence alone. An actuarial analysis of patients with grade 3 STS is shown in Figure 2: a significant advantage for both RFS ($p$=0.03, two-sided) (Fig. 2b) and TDF ($p$=0.03, two-sided) (Fig. 2d) was observed in patients receiving chemotherapy, as compared with patients treated with radiotherapy only. In contrast, Kaplan–Meier plots for TLF ($p$=0.06) (Fig. 2c) and OS ($p$=0.4) (Fig. 2a) did not differ significantly.

*Response to treatment, RFS, TLF, TDF and OS in patients with grade 2 STS.* Considering grade 2 STS, 12 patients with grade 2 STS received radiotherapy only versus six patients who underwent additional chemotherapy. Nine patients (75%) from the control group versus five patients (83%) from the chemotherapy group had no evidence of tumor recurrence. Tumor relapse occurred in three patients (25%) treated with radiotherapy alone (one local failure, two distant failures) versus one (17%) patient who received additional chemotherapy and subsequently developed lung metastases ($p$>0.05). No significant difference regarding RFS, TLF, TDF or OS was observed ($p$>0.05), respectively (plots not shown).

### Discussion

In this study, 59 patients with histopathologically verified STS underwent primary surgery by wide or marginal resection and were subsequently randomized to receive either adjuvant RT alone or, in a combined modality approach, under the inclusion of six adjuvant courses of the IFADIC chemotherapy regimen augmented in time. After a mean observation period of 41 months and an analysis of all included patients, the present trial did not show any significant difference favoring either approach concerning RFS, TLF, TDF or OS. Application of the intensified IFADIC regimen was associated with relatively mild to moderate side effects, with leukopenia and alopecia representing the most common toxicities. As compared with previous studies using doxorubicin-containing combination regimens in advanced STS,[21,32] the frequency of treatment-related grade 3 and 4 toxicities was generally lower in the present study. This might be due to the fact that, in our study, the dose of doxorubicin of 50 mg/m$^2$/cycle was lower, as compared with 60 mg/m$^2$/cycle in other similar combination regimens,[21] concomitant administration of radiotherapy and doxorubicin-containing chemotherapy (i.e. during cycles 3 and 4) was avoided and G-CSF was used on an only mandatory basis. CT was discontinued in two patients (6%) after two cycles due to impairment of wound healing.

**Table 4.** *Responses to treatment in patients with grade 3 soft tissue sarcoma (STS)*

| Reponse rates | RT and CT (*n*=25) | RT (*n*=16) |
|---|---|---|
| No evidence of tumor recurrence (%) | 19 (76%) | 7 (44%) |
| Overall relapses (%) | 6 (24%) | 9 (56%) |
| Local failures (%) | 2 (8%)[1] | 5 (31%)[2] |
| Distant failures (%) | 5 (20%)[1] | 8 (50%)[2] |
| Extent of tumor | | |
| ≥10 cm | 12 (48%) | 7 (44%) |
| <10 cm | 13 (52%) | 9 (56%) |

[1]Including one patient with local relapse and lung metastases.
[2]Including four patients with local relapse and lung metastases.
CT, Chemotherapy; RT, radiotherapy.

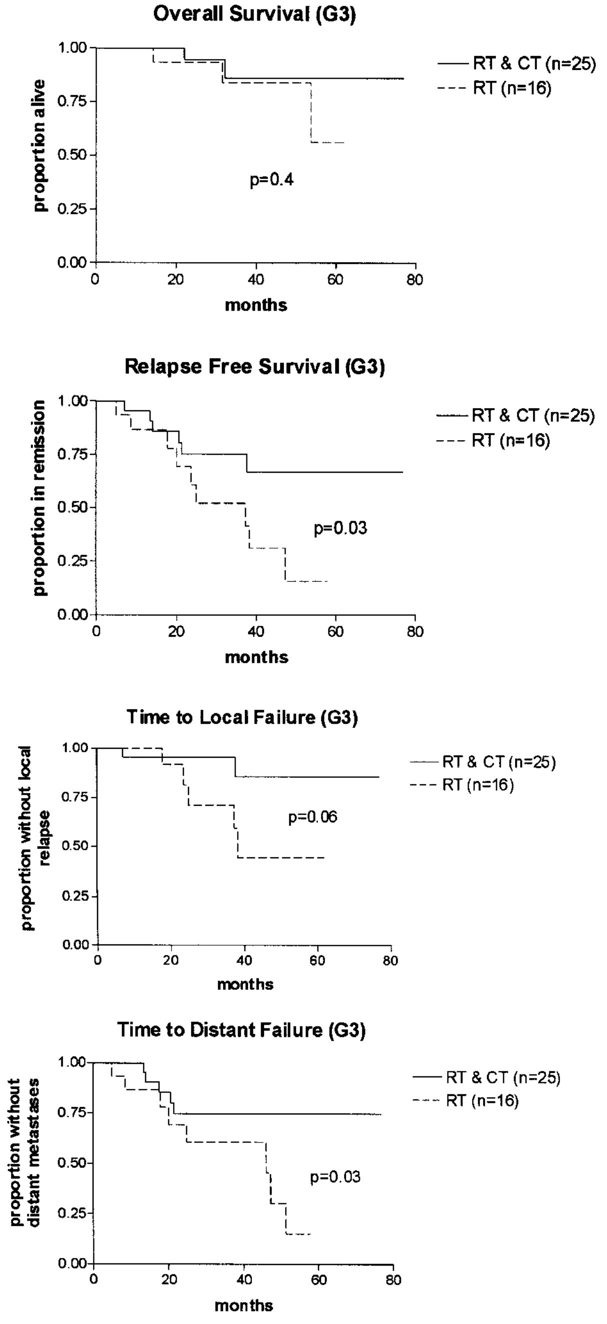

Figure 2.

The mean number of 5.7±1 administered CT cycles, all of which were given in the assigned dose and in a relative dose intensity of 93%, illustrate the feasibility of the chosen regimen. Due to standard anti-emetic medication with 5-$HT_3$-antagonists combined with metoclopramide, no episodes of emesis grades III or IV occurred. Furthermore, none of the patients developed neutropenic sepsis. Most patients received IFADIC on an outpatient basis, thus further stressing the feasibility of the presently described IFADIC regimen.

Despite extensive data on response to CT of patients with metastatic STS,[33] only 13 prospective randomized studies have been performed to combat micrometastases by adjuvant CT following adequate local surgical treatment of STS. Six studies included single-agent doxorubicin in the treatment protocol,[10,11,34–36] whereas five employed combination chemotherapy.[12–16,37] One study was limited to uterine sarcomas.[38] A significant difference upon RFS or OS was reported in only three studies,[10–13] whereas five studies using single-agent doxorubicin in the chemotherapy arm did not show any significant difference in either RFS or OS.[34–38] However, a recently published meta-analysis of 1568 patients[17] provided evidence that adjuvant doxorubicin-based CT significantly improved local, distant and overall recurrence-free survival. In addition, a trend towards improved overall survival was postulated.

The present regimen of augmented adjuvant polychemotherapy was chosen due to recent insights that have shown that polychemotherapy including ifosfamide and doxorubicin (the most active agents in adult STS[19]) exerts higher activity, as compared with monotherapy,[32] and a dose-response relationship for both drugs in advanced or metastatic STS derived from several trials.[39–41] Finally, dose escalation by the addition of other agents extended the multidrug profile at lower dose levels.[42] As only few data exist about the efficacy of augmented CT for the adjuvant treatment of STS, and following the already presented considerations, we have decided to administer the conventional IFADIC regimen augmented in time by an abbreviation of inter-treatment-intervals by G-CSF support.[43] The results of our study indicated, however, that adjuvant RT plus CT led to similar results in RFS, TLF and TDF, as compared with adjuvant RT only and, therefore, that patients with STS fulfilling the employed inclusion criteria derived no benefit from CT

administered in addition to RT. However, a retrospective subgroup analysis of patients with grade 3 STS revealed an advantage for the application of CT versus RT alone and showed evidence of a significant improvement of both RFS and TDF. Tumor relapse occurred in 56% of patients with grade 3 STS from the control arm, as compared with 24% of patients with identical characteristics included in the chemotherapy arm ($p<0.05$). Within this context, 50% of patients from the control arm developed lung metastases, as compared with 20% of patients from the chemotherapy arm ($p=0.04$). However, this retrospective subgroup analysis has to be interpreted with caution due to several reasons. First, patient stratification was not carried out for tumor grade during the randomization process resulting in an imbalance concerning treatment of STS patients with both, grade 2 (12 patients in the control arm versus six in the chemotherapy arm) and grade 3 (25 patients in the chemotherapy arm versus 16 in the control arm) malignancy. Second, the inclusion of more patients with poorer prognosis into the chemotherapy arm could explain the trend towards improved RFS, TLF and TDF observed in this group. Although only a relatively small number of patients with STS was included in the present study, its results are consistent with a meta-analysis favoring adjuvant chemotherapy including anthracycline[17] and, although not equally confident, corroborate results of a study performed by Frustaci *et al.*[13] who demonstrated improved RFS and OS in patients with high-grade STS treated with adjuvant chemotherapy. Adequate tumor staging in STS patients should include chest CT scan. Inclusion criteria of the present study, initiated in 1992, consisted of chest X-ray only due to the scheduled multicenter character of this trial. However, CT scan of the chest was performed in 95% of the patients, as they were included from academic centers or central community hospitals. Currently, all of the remaining 5% of patients are in CR. Thus, a lack of initial staging cannot be held responsible for the current results on relapse. Furthermore, the low local recurrence rate of 6% in the chemotherapy arm may be contributed to the intensified IFADIC regimen. However, this assumption would have to be corroborated within the frame of a randomized trial including a larger number of patients. In contrast, the surprisingly relatively high local relapse rate (21%) in patients receiving RT only probably precludes hyperfractionated accelerated radiotherapy as exclusive adjuvant treatment of high-risk STS patients in future trials.

We thus conclude that the safety profile of intensified IFADIC added to radiotherapy was manageable and tolerable. Patients randomized to receive intensified IFADIC plus radiotherapy did not experience a significant benefit concerning OS, RFS, TLF and TDF, as compared with radiotherapy only. A potential benefit resulting from the administration of adjuvant chemotherapy consisting of intensified IFADIC for patients with grade 3 STS needs to be further validated within the frame of a prospective randomized trial.

### Acknowledgments

The authors are grateful to Dr Günther Nirnberger for the performance of the statistical analysis and to Mrs Veronika Sramek for monitoring centers and patients. This study was supported by grants from the 'Medizinisch-wissenschaftlicher Fonds des Bürgermeisters der Bundeshauptstadt Wien' and 'Kommission Onkologie der Medizinischen Fakultät der Universität Wien', and by a scientific grant from Roche®.

### Appendix*

Members and affiliations of the Austrian Cooperative Soft Tissue Sarcoma Study Group, listed in alphabetic order, are as follows. C. Bosse, Department of Internal Medicine, Hospital Klosterneuburg; W. Dobrowsky, Department of Radiotherapy and Radiobiology, University Hospital; J. Grohs, Department of Orthopedic Surgery, University Hospital; H. Hausmaninger, Department of Internal Medicine, General Hospital, Salzburg; T. Hintringer, Department of Plastic Surgery, Hospital 'Barmherzige Schwestern', Linz, Austria; G. Holzer, Department of Orthopedic Surgery, University Hospital; J. Jagenbrein, Department of Orthopedic Surgery, Orthopedic Hospital Gersthof, Vienna; T. Katterschafka, Department of Orthopedic Surgery, University Hospital; G. Klein, Department of Orthopedic Surgery, University Hospital; W.J. Koestler, M. Krainer, Clinical Division of Oncology/ Department of Medicine I, University Hospital; S. Lang, Department of Clinical Pathology, University Hospital; A. Leithner, Department of Orthopedic Surgery; M. Mühlbauer, Department of Orthopedic Surgery, Orthopedic Hospital Gersthof, Vienna; B. Pokrajac, Department of Radiotherapy and Radiobiology, University Hospital; M. Salzer-Kuntschik, Department of Clinical Pathology, University Hospital; B. Schnack, Clinical Division of Oncology/ Department of Medicine I, University Hospital; S. Spendel, Department of Plastic Surgery Hospital 'Barmherzige Schwestern', Linz, Austria.

### References

1 Gustafson P. Soft tissue sarcoma. Epidemiology and prognosis in 508 patients. *Acta Orthop Scand* 1994; 65(suppl 259):1–31.
2 Mertens WC, Bramwell VH. Adjuvant chemotherapy in the treatment of soft-tissue sarcoma. *Clin Orthop Relat R* 1993; 289:81–93.
3 Mazanet R, Antman KH. Adjuvant therapy for sarcomas. *Semin Oncol* 1991; 18:603–12.
4 Alho A, Alvegard TA, Berlin O, *et al.* Surgical margin in soft tissue sarcoma. The Scandinavian Sarcoma Group experience. *Acta Orthop Scand* 1989; 60:687–92.
5 Blomqvist CP, Holsti LR. Radiotherapy for soft tissue sarcoma. *Acta Oncol* 1989; 28(suppl 2):37–9.

6 Suit HD, Mankin HJ, Wood WC, *et al.* Treatment of the patient with stage M0 soft tissue sarcoma. *J Clin Oncol* 1988; 6:854–62.
7 Suit HD. The George Edelstyn Memorial Lecture. Radiation in the management of malignant soft tissue tumours. *Clin Oncol* 1989; 1:5–10.
8 Dunst J, Jurgens H, Sauer R, *et al.* Radiation therapy in Ewing's sarcoma: an update of the CESS 86 trial. *Int J Radiat Oncol Biol Phys* 1995; 32:919–30.
9 Rosenberg SA, Suit HD, Baker LH. Sarcomas of soft tissues. In: De Vita VT, Hellman S, Rosenberg SA, eds. *Cancer: Principles and Practice of Oncology.* Philadelphia: J.B. Lippincott, 1985.
10 Gherlinzoni F, Bacci G, Picci P, *et al.* A randomized trial for the treatment of high-grade soft-tissue sarcomas of the extremities: preliminary observations. *J Clin Oncol* 1986; 4:552–8.
11 Gherlinzoni F, Picci P, Bacci G, *et al.* Late results of a randomized trial for the treatment of soft tissue sarcomas of the extremities in adult patients [abstract 1633]. *Proc Am Soc Clin Oncol* 1993; 12:468.
12 Ravaud A, Nguyen BB, Coindre JM, *et al.* Adjuvant chemotherapy with CYVADIC in high-risk soft tissue sarcoma: a randomized prospective trial. In: Salmon SE, ed. *Adjuvant Therapy of Cancer VI.* Philadelphia: W.B. Saunders, 1990:556–66.
13 Frustaci S, Gherlinzoni F, De Paoli A, *et al.* Preliminary results of an adjuvant randomized trial on high risk extremity soft tissue sarcomas. The interim analysis [abstract 1785]. *Proc Am Soc Clin Oncol* 1997; 16:496a.
14 Bramwell V, Rouesse J, Steward W, *et al.* Adjuvant CYVADIC chemotherapy for adult soft tissue sarcoma-reduced local recurrence but no improvement in survival: a study of the European Organization for Research and Treatment of Cancer Soft Tissue and Bone Sarcoma Group. *J Clin Oncol* 1994; 12:1137–49.
15 Chang A, Kinsella T, Glatstein E, *et al.* Adjuvant chemotherapy for patients with high-grade soft-tissue sarcomas of the extremities. *J Clin Oncol* 1988; 6:1491–500.
16 Benjamin TO, Terjanian TO, Fenoglio CJ, *et al.* The importance of combination chemotherapy for adjuvant treatment of high-risk patients with soft-tissue sarcomas of the extremities. In: Salmon SE, ed. *Adjuvant Therapy of Cancer.* Orlando: Grune and Stratton, 1987:735–44.
17 Sarcoma Meta-Analysis Collaboration. Adjuvant chemotherapy for localised resectable soft-tissue sarcoma of adults: meta-analysis of individual data. *Lancet* 1997; 350:1647–54.
18 Yap B, Baker LH, Sinkovics JG, *et al.* Cyclophosphamide, vincristine, adriamycin and DTIC (CYVADIC) combination chemotherapy for the treatment of advanced sarcomas. *Cancer Treat Rep* 1980; 64:93.
19 Antman KH, Elias AD. Chemotherapy of advanced soft-tissue sarcomas. *Semin Surg Oncol* 1988; 4:53–8.
20 Elias A, Ryan L, Sulkes A, *et al.* Response to mesna, doxorubicin, ifosfamide and dacarbazine in 108 patients with metastatic or unresectable sarcoma and no prior chemotherapy. *J Clin Oncol* 1989; 7:1208–16.
21 Antman K, Crowley J, Balcerzak SP, *et al.* An intergroup phase III randomized study of doxorubicin and dacarbazine with or without ifosfamide and mesna in advanced soft tissue and bone sarcomas. *J Clin Oncol* 1993; 11:1276–85.
22 Frei E III, Canellos GP. Dose: a critical factor in cancer chemotherapy. *Am J Med* 1980; 69:585–94.
23 Savarese DM, Hsieh C, Stewart FM. Clinical impact of chemotherapy dose escalation in patients with hematologic malignancies and solid tumors. *J Clin Oncol* 1997; 15:2981–95.
24 Patel SR, Vadhan-Raj S, Burgess MA, *et al.* Dose intensive therapy does improve response rates—updated results of studies of adriamycin and ifosfamide with growth factors in patients with untreated soft tissue sarcomas [abstract 1794]. *Proc Am Soc Clin Oncol* 1997; 16:499a.
25 Nielsen OS, Judson I, Hoesel v Q, *et al.* Effect of high dose ifosfamide in advanced soft tissue sarcomas. A multicenter phase II study of the EORTC soft tissue and bone sarcoma group [abstract 1992]. *Proc Am Soc Clin Oncol* 1998; 17:517a.
26 Williams SD. High dose therapy in germ cell tumors: when, what, and how much? *Ann Oncol* 1992; 3:780–1.
27 Enneking WF. Staging of musculoskeletal neoplasms. In: *Current Concepts of Diagnosis and Treatment of Bone and Soft Tissue Tumors.* Heidelberg: Springer Verlag, 1984.
28 Enneking WF. *Muskuloskeletal Tumor Surgery* vols I, II. New York: Churchill Livingstone, 1983.
29 Enzinger FM, Weiss SW. *Soft Tissue Tumors* 3rd edition. St. Louis: Mosby-Year Book, Inc., 1995.
30 Fletcher CDM. *Diagnostic Histopathology of Tumors* vols I and II, 1st edition. Hong Kong: Churchill Livingstone, 1995.
31 Le Cesne A, Judson I, Crowther D, *et al.* Randomized phase III study comparing conventional-dose doxorubicin plus ifosfamide versus high-dose doxorubicin plus ifosfamide plus recombinant human granulocyte-macrophage colony-stimulating factor in advanced soft tissue sarcomas: a trial of the European Organization for Research and Treatment of Cancer/Soft Tisue and Bone Sarcoma Group. *J Clin Oncol* 2000; 18:2676–84.
32 Edmonson J, Ryan L, Blum R, *et al.* Randomized comparison of doxorubicin alone versus ifosfamide plus doxorubicin or mitomycin, doxorubicin, and cisplatin against advanced soft tissue sarcomas. *J Clin Oncol* 1993; 11:1269–75.
33 Glabbeke v M, Oosterom v AT, Oosterhuis JW, *et al.* Prognostic factors for the outcome of chemotherapy in advanced soft tissue sarcoma: an analysis of 2,185 patients treated with anthracycline-containing first-line regimens—a European Organization for Research and Treatment of Cancer Soft Tissue and Bone Sarcoma Group Study. *J Clin Oncol* 1999; 17:150–57.
34 Alvegard TA, Sigurdsson H, Mouridsen H, *et al.* Adjuvant chemotherapy with doxorubicine in high-grade soft tissue sarcoma: a randomized trial of the Scandinavian Sarcoma Group. *J Clin Oncol* 1989; 7:1504–13.
35 Antman K, Ryan L, Borden E, *et al.* Pooled results from three randomized adjuvant studies of doxorubicin versus observation in soft tissue sarcoma: 10 year results and review of the literature. In: Salmon SE, ed. *Adjuvant Therapy of Cancer VI.* Philadelphia: W.B. Saunders, 1990:529–43.
36 Eilber FR, Giuliano AE, Huth JF, *et al.* A randomized prospective trial using postoperative adjuvant chemotherapy (adriamycin) in high-grade extremity soft tissue sarcoma. *Am J Clin Oncol* 1988; 11:39–45.
37 Edmonson JH, Fleming TR, Ivins JC, *et al.* Randomized study of systemic chemotherapy following complete excision of nonosseous sarcomas. *J Clin Oncol* 1984; 2:1390–6.
38 Omura GA, Blessing JA, Major F, *et al.* A randomized clinical trial of adjuvant adriamycin in uterine sarcomas: a gynecologic oncology group study. *J Clin Oncol* 1985; 3:1240–5.
39 Steward WP, Verweij J, Somers R, *et al.* Granulocyte-macrophage colony-stimulating factor allows safe escalation of dose-intensity of chemotherapy in metastatic adult soft tissue sarcomas: a study of the European Organization for Research and Treatment of Cancer Soft Tissue and Bone Sarcoma Group. *J Clin Oncol* 1993; 11:15–21.

40 Benjamin RS, Legha SS, Patel SR, *et al.* Single-agent ifosfamide studies in sarcomas of soft tissue and bone: the MD Anderson experience. *Cancer Chemoth Pharm* 1993; 31(suppl 2):174.
41 O'Bryan RM, Baker LH, Gottlieb JE, *et al.* Dose response evaluation of adriamycin in human neoplasia. *Cancer* 1977; 39:1940.
42 DeVita VT. Dose-response is alive and well. *J Clin Oncol* 1986; 4:1157–9.
43 Bui BN, Chevallier B, Chevreau C, *et al.* Efficacy of lenograstim on hematologic tolerance to MAID chemotherapy in patients with advanced soft tissue sarcoma and consequences on treatment dose-intensity. *J Clin Oncol* 1995; 13:2629–36.
